# Targeting glioma stem cells *in vivo* by a G-quadruplex-stabilizing synthetic macrocyclic hexaoxazole

**DOI:** 10.1038/s41598-017-03785-8

**Published:** 2017-06-15

**Authors:** Takahiro Nakamura, Sachiko Okabe, Haruka Yoshida, Keisuke Iida, Yue Ma, Shogo Sasaki, Takao Yamori, Kazuo Shin-ya, Ichiro Nakano, Kazuo Nagasawa, Hiroyuki Seimiya

**Affiliations:** 1 Division of Molecular Biotherapy, Cancer Chemotherapy Center, Japanese Foundation for Cancer Research, Tokyo, 135-8550 Japan; 2 Department of Biotechnology and Life Science, Faculty of Engineering, Tokyo University of Agriculture and Technology, Tokyo, 184-8588 Japan; 3 Molecular Chirality Research Center, Synthetic Organic Chemistry, Department of Chemistry, Graduate School of Science, Chiba University, Chiba, 263-8522 Japan; 4 Division of Molecular Pharmacology, Cancer Chemotherapy Center, Japanese Foundation for Cancer Research, Tokyo, 135-8550 Japan; 5 Department of Life Science and Biotechnology, Biotechnology Research Institute for Drug Discovery, National Institute of Advanced Industrial Science and Technology, Tokyo, 135-0064 Japan; 6 Department of Neurosurgery and Comprehensive Cancer Center, University of Alabama at Birmingham, Birmingham, AL 35294 USA; 7 Pharmaceuticals and Medical Devices Agency, Tokyo, 100-0013 Japan

## Abstract

G-quadruplex (G4) is a higher-order nucleic acid structure that is formed by guanine-rich sequences. G4 stabilization by small-molecule compounds called G4 ligands often causes cytotoxicity, although the potential medicinal impact of this effect has not been fully established. Here we demonstrate that a synthetic G4 ligand, Y2H2-6M(4)-oxazole telomestatin derivative (6OTD), limits the growth of intractable glioblastoma (grade IV glioma) and glioma stem cells (GSCs). Experiments involving a human cancer cell line panel and mouse xenografts revealed that 6OTD exhibits antitumor activity against glioblastoma. 6OTD inhibited the growth of GSCs more potently than it did the growth of differentiated non-stem glioma cells (NSGCs). 6OTD caused DNA damage, G1 cell cycle arrest, and apoptosis in GSCs but not in NSGCs. These DNA damage foci tended to colocalize with telomeres, which contain repetitive G4-forming sequences. Compared with temozolomide, a clinical DNA-alkylating agent against glioma, 6OTD required lower concentrations to exert anti-cancer effects and preferentially affected GSCs and telomeres. 6OTD suppressed the intracranial growth of GSC-derived tumors in a mouse xenograft model. These observations indicate that 6OTD targets GSCs through G4 stabilization and promotion of DNA damage responses. Therefore, G4s are promising therapeutic targets for glioblastoma.

## Introduction

While innovative therapeutic strategies for many cancers have been established, effective treatment of glioblastoma multiforme (GBM)—grade IV glioma and the most prevalent primary brain tumor in adults—remains challenging^1–5^. Current standard therapy involves temozolomide (TMZ) treatment and radiation therapy following surgical resection of the primary lesion, but outcomes are generally poor (approximate median overall survival time of 12–15 months after diagnosis)^1–6^. Therefore, novel approaches to GBM treatment are required. GBM exhibits intratumor heterogeneity, with glioma stem cells (GSCs) and non-stem glioma cells (NSGCs) both present in lesions^7–10^. GSCs are considered responsible for tumor propagation, resistance to chemotherapy and radiation therapy, and disease recurrence^7–10^. Therefore, GSCs represent a promising therapeutic target for GBM.

**Figure 1:**
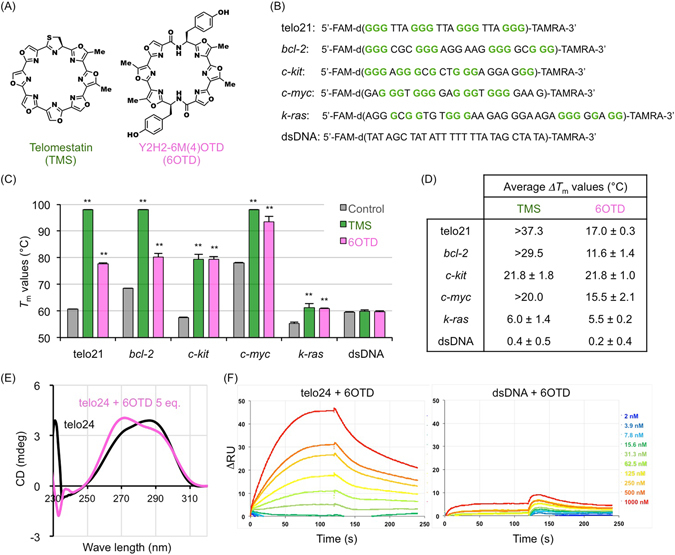
6OTD stabilizes G4 structures. (**A**) Chemical structures of telomestatin (TMS) and 6OTD. (**B**) List of oligonucleotides used in the FRET melting assay. All oligonucleotides are dual-labeled with FAM and TAMRA at their 5′ and 3′ ends, respectively. Guanines involved in G4 formation are colored in green. (**C**) *T*_m_ values of the oligonucleotides (0.2 μM) determined by the FRET melting assay in the absence or the presence of G4 ligands (1.0 μM) in K^+^-rich buffer. Each *T*_m_ value represents the mean of triplicate assays (**P < 0.01). In the presence of TMS, *T*_m_ values of telo21, *bcl*-*2*, and *c*-*myc* exceeded 98 °C, which was the highest temperature of this measurement. *Error bars*, standard deviation (SD). (**D**) *ΔT*_m_ values for TMS and 6OTD against each nucleotide. Each value represents the mean ± SD of triplicate assays. (**E**) CD spectra of the telomeric 5′-d[TTAGGG]_4_-3′ (telo24) oligonucleotide in the presence of 100 mM KCl at 25 °C. Concentrations of telo24 and 6OTD were 10 μM and 50 μM, respectively. (**F**) SPR analysis of telo24 and dsDNA with 6OTD. K_D_ value for the telo24-6OTD interaction was 163.7 ± 29.3 nM, determined by three independent experiments. K_D_ value for the dsDNA-6OTD could not be determined due to the low affinity of the molecules.

We have previously shown that telomestatin (TMS; Fig. 1A), a natural small-molecule compound isolated from *Streptomyces anulatus* 3533-SV4^11^, has a preferential anti-proliferative effect on GSCs *in vitro* and *in vivo* ^12^. TMS stabilizes G-quadruplexes (G4s)^13^, which are non-canonical steric structures formed by guanine-rich nucleic acids. G4s are present in telomeres and the promoter regions of various cancer-related genes^12, 14–19^. Consistent with the broad distribution of G4s, TMS has multiple modes of action against GSCs. First, TMS causes telomere dysfunction, which is associated with dissociation of telomeric repeat-binding factor 2 (TRF2) from telomeres in cancer cells^20^. Second, TMS induces a higher level of the replication stress response in GSCs than in NSGCs^21^. Third, TMS downregulates the expression of *c*-*Myb*, a proto-oncogene that is strongly expressed by GSCs—but not NSGCs—at both the mRNA and protein levels. This effect probably results from G4 stabilization on the promoter region of *c*-*Myb* ^12^. These observations explain the selective antitumor effect of TMS on GSCs and suggest that G4s are promising molecular targets for treatment of GBM.

While TMS is one of the most potent G4 ligands, several concerns surround its medicinal application. First, only a small amount of TMS can be obtained from a large culture of *Streptomyces anulatus* 3533-SV4, presenting difficulties for extensive *in vivo* studies. Second, TMS is neither stable nor highly soluble in aqueous solution. Therefore, we have developed a series of synthetic oxazole TMS derivatives (OTDs) that are chemically stable, potently stabilize telomeric G4s, and can be synthesized on a large scale^22–37^. Among these compounds, Y2H2-6M(4)OTD (described as 6OTD hereafter in this manuscript; Fig. 1A) exerts a significant antiproliferative activity on several cancer cell lines *in vitro* ^31, 32^. However, whether this compound elicits DNA damage response in cancer cells and exhibits antitumor efficacy *in vivo* has remained unknown.

Here we demonstrate that 6OTD inhibits the growth of human glioblastoma and glioma stem cell lines *in vitro* and *in vivo*. We found that 6OTD potently and selectively stabilized several G4s, including those in telomeres and in the promoters of some oncogenes. Consistently, 6OTD induced a DNA damage response at G4-forming sequences, promoting cell cycle arrest and apoptosis in GSCs. These observations may facilitate development of innovative therapeutic drugs against intractable brain tumors.

## Results

### 6OTD stabilizes telomeric and non-telomeric G4s

We conducted a fluorescence resonance energy transfer (FRET) melting assay^38, 39^ to evaluate the ability of 6OTD to stabilize G4s and investigate the specificity of these interactions. We used five different G4-forming oligonucleotides (GFOs) which have been described previously [telomeric GFO (telo21) and non-telomeric, oncogene-associated GFOs (*bcl*-*2*, *c*-*kit*, *c*-*myc*, *k*-*ras*)]^32^. As a negative control for specificity validation, a non-GFO that adopts a double-stranded configuration through hairpin formation was also included in the assay (Fig. 1B). The *T*_m_ values of these oligonucleotides in the presence of 60 mM KCl—which is required for G4 formation—and 1.0 μM 6OTD or TMS as a positive control for G4 stabilization were measured. Figure 1C and D show the *ΔT*_m_ value of each condition. These assays revealed that 6OTD and TMS stabilize all GFOs but not the hairpin-forming non-GFO. While TMS exhibited higher *ΔT*_*m*_ values than 6OTD against telo21, *bcl*-*2*, and *c*-*myc* GFOs, these two compounds showed comparable capacities to stabilize *c*-*kit* and *k*-*ras* G4s. Samples with TMS contained a low level (0.02%) of methanol, which did not affect the results (data not shown). We performed the assay in the absence or the presence of 0.5% DMSO, which gave *Tm* values (Telo21) of 60.0 ± 0.2 °C and 59.9 ± 0.0 °C, respectively. We also performed the same assay with non-GFO in the absence or the presence of 0.5% DMSO, which gave *Tm* values of 60.1 ± 0.3 °C and 59.5 ± 0.1 °C, respectively. Thus, we consider that such low concentration of DMSO has no or only minimal effect, if any, on the stability of the higher-order nucleic acid structures examined in this study.

By performing the electrophoretic mobility shift assay (EMSA), we have already demonstrated that 6OTD binds the telomeric 5′-d[TTAGGG]_4_-3′ oligonucleotide^31^. Furthermore, circular dichroism (CD) spectrum melting assay revealed that *T*_*m*_ values of telo24 GFO in the absence and the presence of 5 eq. 6OTD were 60.1 °C ± 0.0 °C and 66.2 °C ± 0.2 °C, respectively (Fig. 1E and data not shown). Surface plasmon resonance (SPR) analysis also confirmed G4-binding properties of the compound (Fig. 1F).

### 6OTD exhibits antitumor activity against glioblastoma cells *in vitro* and *in vivo*

**Figure 2:**
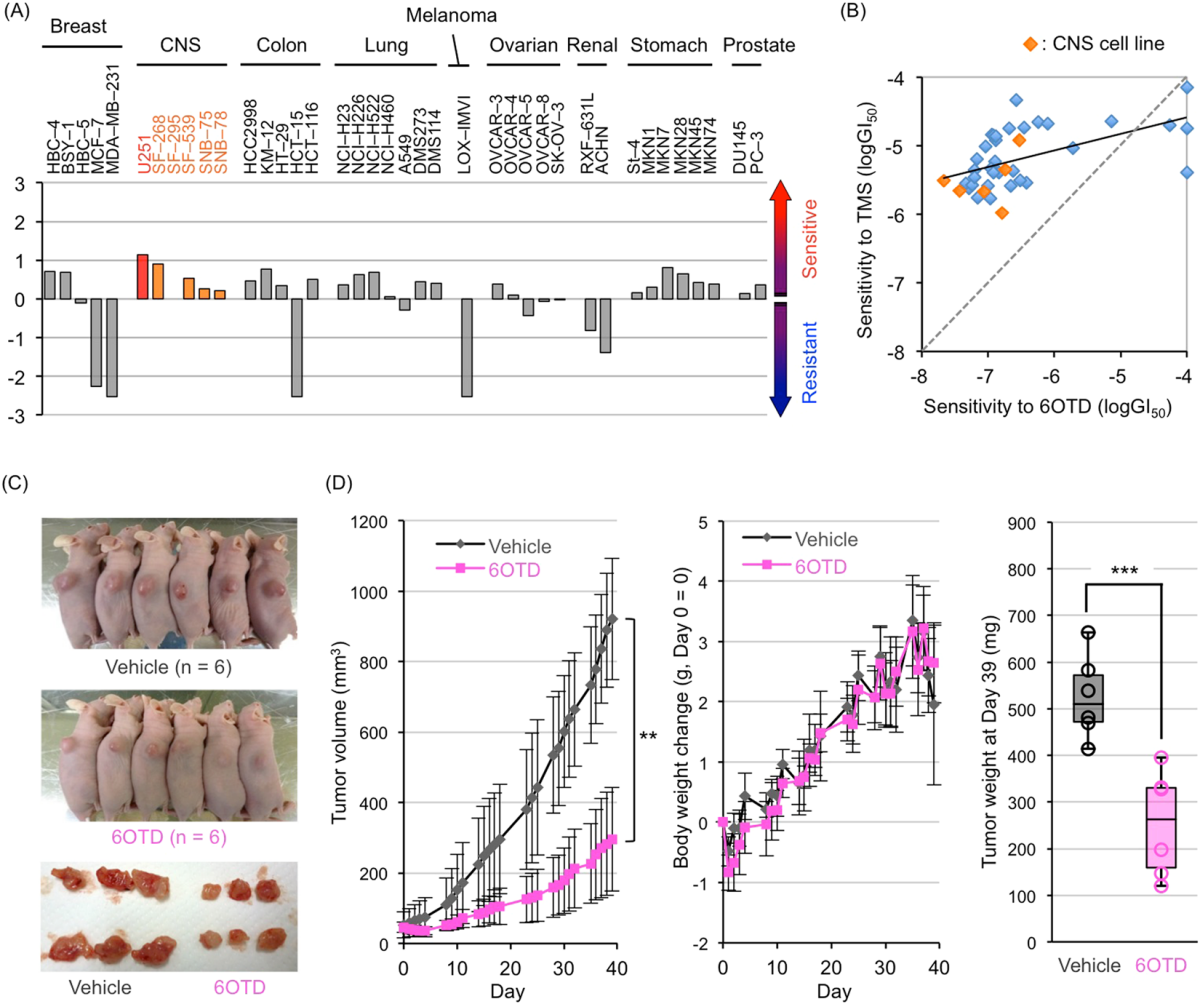
6OTD inhibits the growth of glioma U251 cells *in vitro* and *in vivo*. (**A**) Growth inhibitory effect of 6OTD on the JFCR39 human cancer cell line panel. *Zero* in the vertical axis means the mean midpoint value of log [GI_50_] of 6OTD for all the cell lines, and each *bar* means deviation of the log [GI_50_] for each cell line from the mean midpoint value. Thus, cell lines that were relatively sensitive and resistant to 6OTD are indicated with the vertical bars above and below 0, respectively. CNS: central nervous system. Because the GI_50_ value of SF-295 cells (290 nM) was exactly the same as the average value of all cell lines, the bar for this cell line cannot be seen in the graph (the value is zero). (**B**) Relationship between sensitivities of the cell lines to telomestatin (TMS) and 6OTD. *Black solid line* means linear approximation curve for the 39 data plots. *Orange* and *blue* symbols indicate CNS- and other organ-derived cancer cell lines, respectively. (**C**) Antitumor effect of 6OTD on U251 xenografts. Photos of vehicle (*top*) and 6OTD (*center*)-treated mice at day 35 (n = 6) are shown. *Bottom*, dissected tumors after drug treatment. (**D**) Tumor volume (*left*), body weight change (*center*), and tumor weight at day 39 (*right*) in (**C**). Day 0 means the starting day of drug administration. Error bars mean SD. **P < 0.01, ***P < 0.005.

After confirming the potent and specific G4-stabilizing properties of 6OTD, we next investigated the growth inhibitory potential of this compound against the JCFR39 human cancer cell line panel^40^. Thirty-nine human cancer cell lines derived from various tissues were incubated with increasing concentrations of 6OTD for 48 h, and each GI_50_ value (concentration required for 50% growth inhibition) was calculated (Supplementary Table S1). 6OTD exhibited differential growth inhibitory activity against the 39 cell lines (Fig. 2A). Among them, 27 cell lines had lower GI_50_ values than the average of all cell lines. Especially, 5 of 6 central nervous system (CNS) cancer cell lines were more sensitive to the compound than the average of all cell lines, with GI_50_ values ranging from 21 nM (U251 cells) to 180 nM (SNB-78 cells). TMS also exerts CNS-selective anti-cancer activity in the range of 1 to 10 μM. The two compounds shared moderately similar patterns of cell specificity (r = 0.533, *P* < 0.001) (Fig. 2B). The mean GI_50_ value of 6OTD for 39 cell lines was 0.30 μM, whereas that of TMS was 6.5 μM. Thus, 6OTD inhibits the *in vitro* growth of human cancer cell lines, especially those from the CNS, more potently than does TMS.

We next examined the therapeutic impact of 6OTD *in vivo* by evaluating the antitumor activity of this compound against human glioblastoma U251 cells subcutaneously injected into nude mice. Eighteen days after xenotransplantation of cells into the right flank of mice, mice were randomized into vehicle control and 6OTD groups (n = 6/group). Vehicle [10% dimethyl sulfoxide (DMSO) in saline, v/v] or 6OTD (240 mg/kg) was administered intraperitoneally five days a week with tumor volumes and body weight recorded. All mice were sacrificed 39 days after beginning drug treatment, and the weights of excised tumors were measured. 6OTD caused significant inhibition of tumor growth (treatment/control: T/C% = 33%) and tumor weights (T/C% = 61%) without any significant effect on body weight (Fig. 2C and D). These data indicate that 6OTD exerts potent antitumor activity against U251 cells *in vitro* and *in vivo*.

### 6OTD preferentially inhibits cell growth and induces apoptosis in GSCs

**Figure 3:**
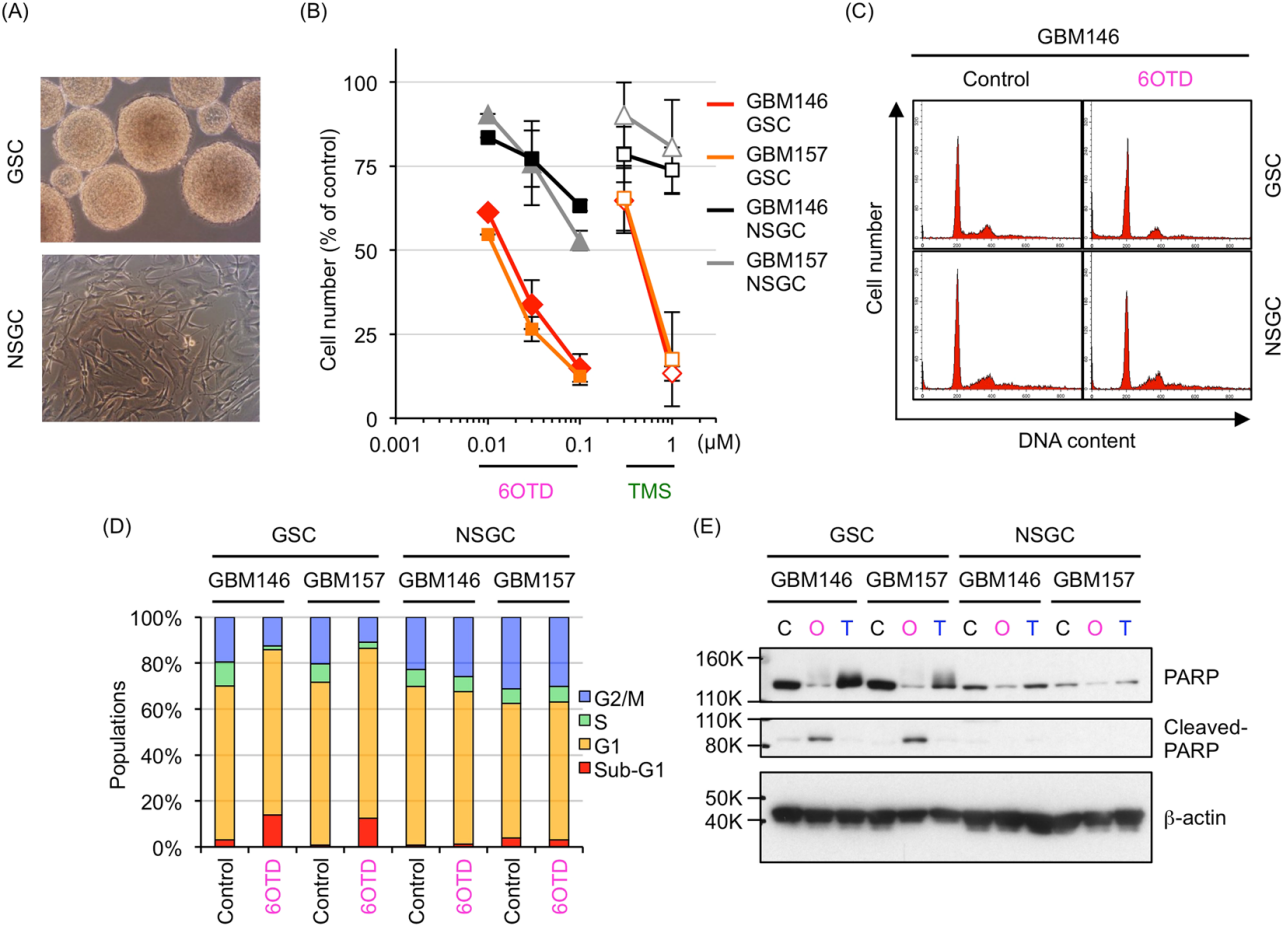
6OTD preferentially targets glioma stem cells rather than non-stem glioma cells. (**A**) Representative photos of glioma stem cells (GSCs) and non-stem glioma cells (NSGCs). GSCs were differentiated into NSGCs by serum stimulation for 7 days^12, 21^. (**B**) Effects of 6OTD and TMS on the growth of two patient-derived GSCs and NSGCs. Cells were treated with indicated concentrations of the compounds for 6 days. Graphs indicate the mean ± SD of triplicate assays. (**C**) Cell-cycle analysis of GSCs and NSGCs treated with 6OTD (0 or 100 nM, 5 days) by flow cytometry with propidium iodide (PI) staining, with the number of cell (*x*-axis) and DNA content (*y*-axis). Indicated histograms are the representative results of duplicate experiments. (**D**) Quantitation of the cell-cycle distribution of control and 6OTD-treated GSCs and NSGCs in (**C**). (**E**) Detection of PARP cleavage in GSCs and NSGCs. Cells were lysed 5 days after DMSO, 6OTD (100 nM), or TMZ (10 μM) treatment, and the resulting lysates were subjected to western blot analysis with the indicated primary antibodies. C: control, O: 6OTD, T: TMZ. Full-length blots are presented in Supplementary Figure S1.

TMS preferentially induces apoptosis in GSCs rather than in differentiated NSGCs. Therefore, we next examined the growth inhibitory activity of 6OTD against GSCs derived from GBM146 and GBM157 cells and their differentiated NSGCs (GSCs differentiate into NSGCs upon treatment with serum for seven days, Fig. 3A). We also compared the antiproliferative effect of 6OTD on GSCs and NSGCs with that of TMS. GSCs and NSGCs were treated with various concentrations of 6OTD or TMS for 6 days. 6OTD inhibited GSC growth more potently than TMS, and GSCs were more sensitive to 6OTD than NSGCs (Fig. 3B). We then evaluated the effects of 6OTD on GSC and NSGC cell cycles by conducting propidium iodide (PI) staining 5 days after drug treatment. 6OTD decreased the proportion of GSCs in S and G2/M phases and increased the proportion in sub-G1 phase. These effects were not observed in NSGCs (Fig. 3C and D). Furthermore, 6OTD triggered cleavage of poly(ADP-ribose) polymerase (PARP), a hallmark of apoptosis, in GSCs but not in NSGCs. Meanwhile, neither GSCs nor NSGCs underwent apoptosis upon treatment with TMZ, a clinical DNA-alkylating agent used for treatment of glioma, at concentrations up to 10 μM (Fig. 3E and Supplementary Fig. S1). These observations indicate that 6OTD can induce *in vitro* cell growth arrest and apoptosis in a GSC-selective manner.

### 6OTD activates DNA damage responses preferentially in GSCs

**Figure 4:**
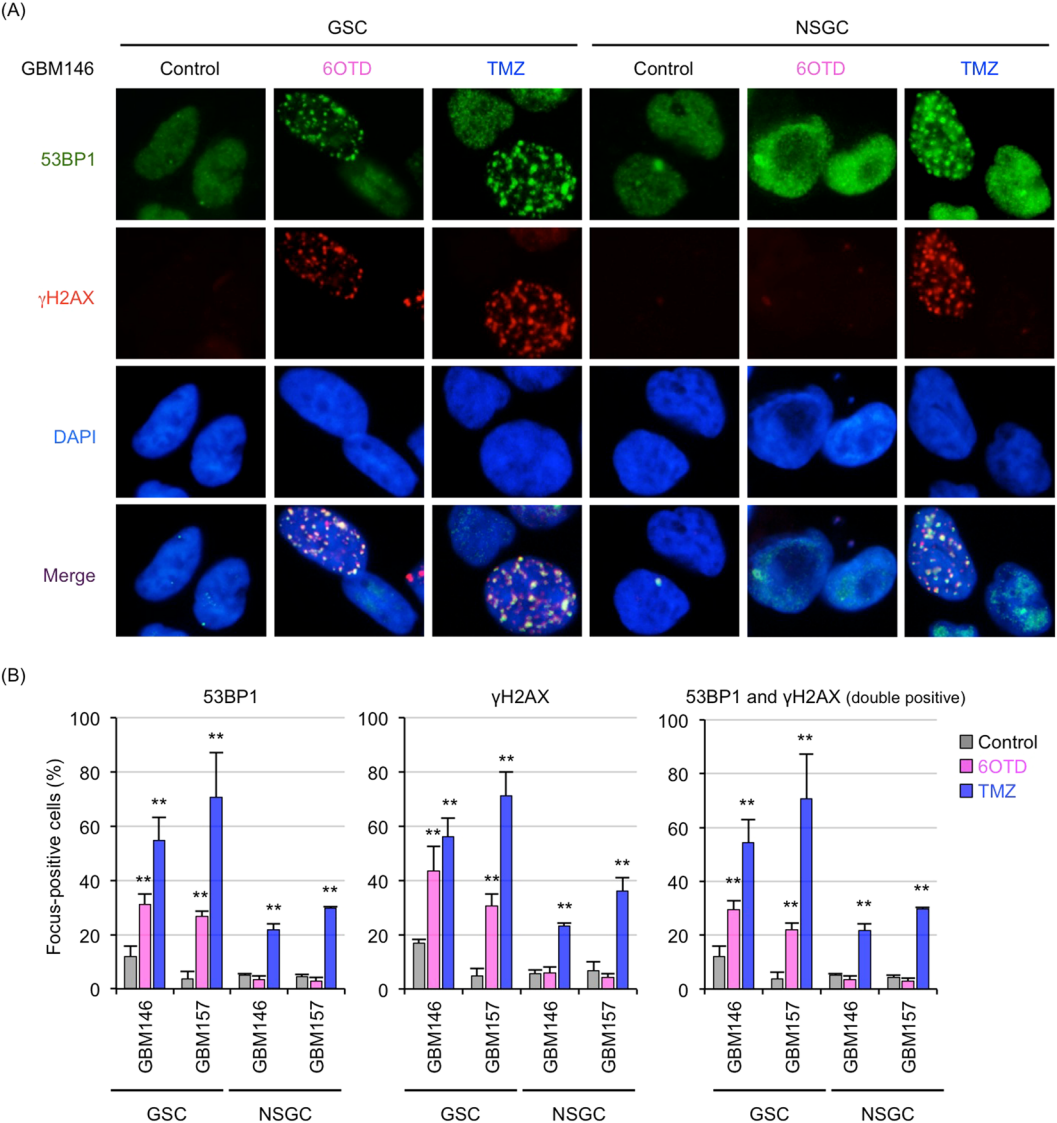
6OTD induces a GSC-selective DNA damage response. (**A**) Immunofluorescence staining of GBM146 (GSCs and NSGCs) exposed to DMSO, 6OTD (100 nM), or TMZ (10 μM) for 3 days. Cells were subjected to immunofluorescence staining with anti-53BP1 (green) and γH2AX (red) antibodies. Nuclear DNA was visualized by DAPI (4′,6-diamidino-2-phenylindole, blue). (**B**) Quantitative analyses of the ratio of 53BP1 (left), γH2AX (middle), and 53BP1/γH2AX double-positive (right) cells in (**A**). At least 200 cells were analyzed for each condition (**P < 0.01). Each bar represents the mean ± SD.

Several G4 ligands, including TMS, activate telomeric and non-telomeric DNA damage responses in multiple cancer cell lines because of the replication stress and genomic instability caused by G4 stabilization^12, 21, 41–43^. We therefore conducted immunofluorescence staining on GSCs and NSGCs treated with 6OTD or TMZ to confirm whether 6OTD elicits DNA damage response in these cells. After treatment with DMSO (control), 6OTD, or TMZ for 3 days, cells were fixed and stained for the DNA damage markers phosphorylated histone H2AX (γH2AX), an early DNA damage marker, and 53BP1, which promotes DNA repair by non-homologous end joining^44, 45^. Cells with more than three DNA damage foci in the nucleus were classified as DNA damage-positive, and we calculated the ratio of these cells in each condition. Few nuclear foci of γH2AX and 53BP1 were observed in the control GSCs (Fig. 4A and Supplementary Fig. S2). 6OTD drastically activated DNA damage response of GSCs. In contrast, 6OTD-treated NSGCs had levels of DNA damage foci similar to those of control cells. Meanwhile, TMZ induced a comparable DNA damage response in both GSCs and NSGCs (Fig. 4B–D). These results indicate that 6OTD induces DNA damage preferentially in GSCs but not NSGCs, although both cells retain the ability to activate the DNA damage response pathway upon treatment with TMZ.

**Figure 5:**
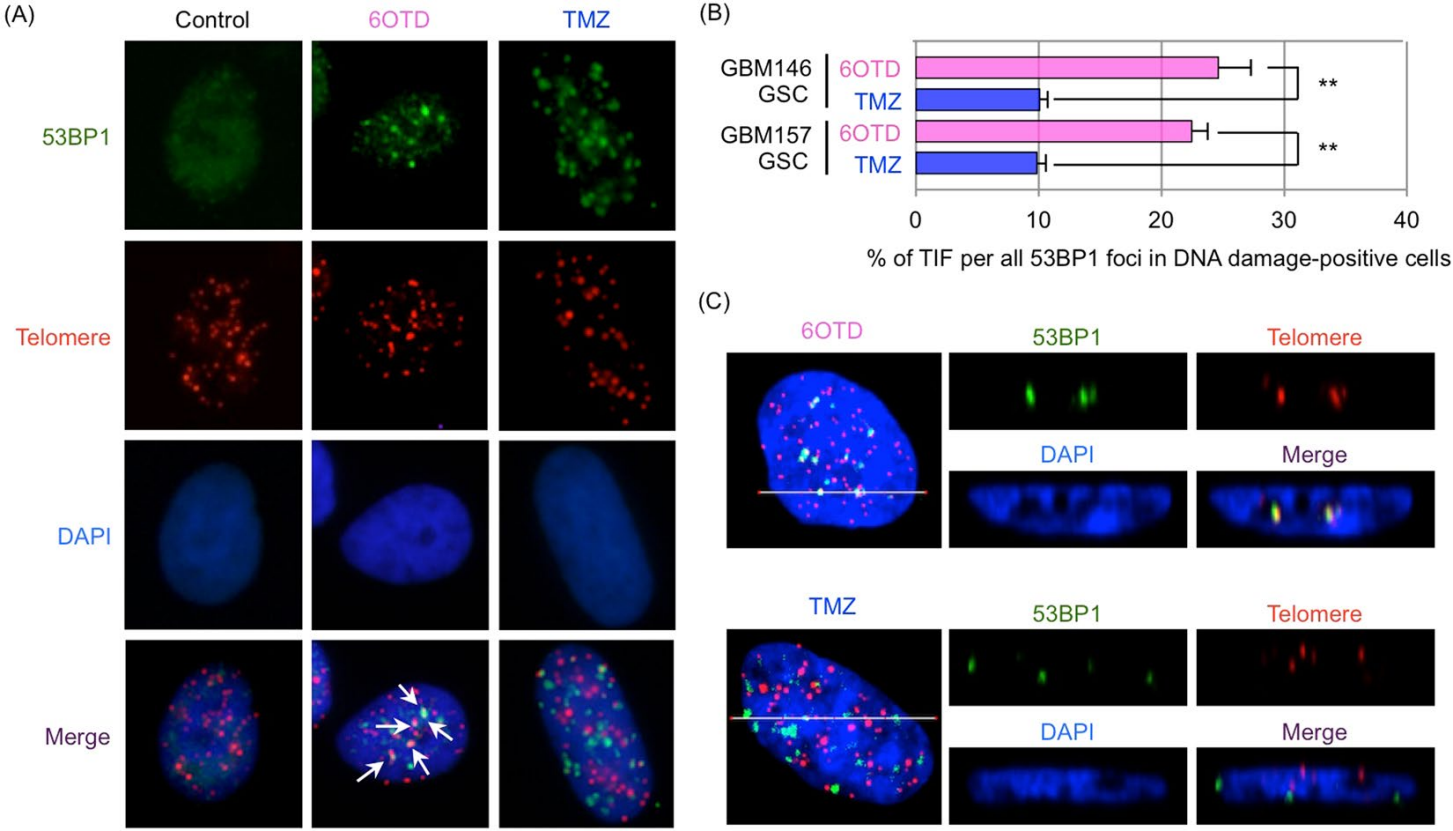
6OTD preferentially attacks telomeric DNA in GSCs. (**A**) iFISH analysis for evaluating telomere dysfunction-induced foci (TIF) in GBM146 cells (GSCs) treated with DMSO, 6OTD (100 nM), or TMZ (10 μM) for 3 days. Cells were labeled with anti-53BP1 antibody and PNA probe for G-rich telomeric DNA. White arrows indicate TIF. (**B**) Quantitative analyses of the percentage of TIF in all 53BP1 foci in DNA damage-positive cells. At least 100 cells were analyzed for each condition. Each bar represents the mean ± SD (**P < 0.01). (**C**) *Z*-stack image of 6OTD (upper) and TMZ (lower)-treated GSCs and consecutive *xz* images on white lines of those cells.

We then conducted immunofluorescence *in situ* hybridization (iFISH) analysis of GSCs treated with 6OTD or TMZ for 3 days to estimate the ratio of telomere dysfunction-induced foci (TIF). In this experiment, we calculated the percentages of 53BP1 foci at telomeres, i.e., TIF, out of all foci in DNA damage-positive cells. Because 6OTD stabilizes G4 DNA selectively (Fig. 1) and telomeres have tandem G4 forming motifs, we speculated that more TIF should be present in 6OTD-treated GSCs compared with TMZ-treated cells. Approximately 10% of 53BP1 foci colocalized with telomeres in DNA damage-positive cells following TMZ treatment. Meanwhile, 22–25% of damage foci colocalized with telomeres in 6OTD-treated GSCs (Fig. 5A–C and Supplementary Fig. S3). These observations suggest that 6OTD recognizes G4s, where it causes GSC-selective DNA damage.

### Anticancer effect of 6OTD against GSCs in intracranially xenografted mice

**Figure 6:**
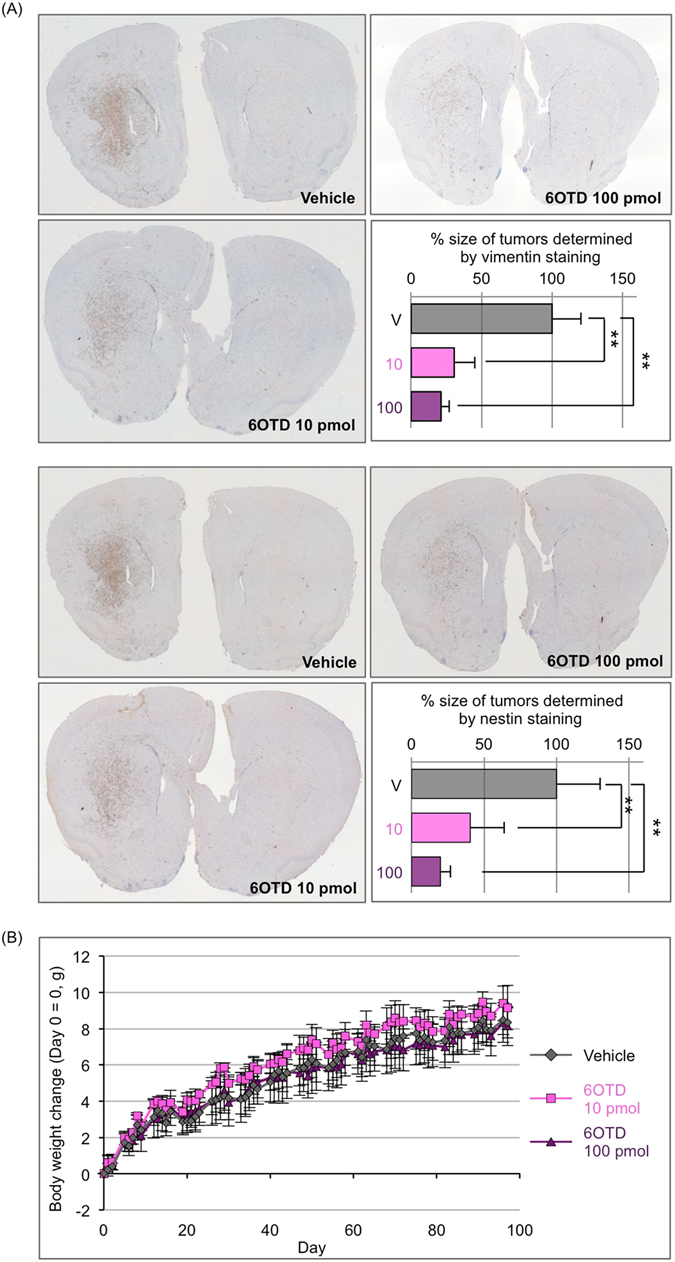
6OTD suppresses GSC growth *in vivo*. (**A**) Effect of 6OTD on intracranial growth of GSCs in a mouse xenograft model. Each mouse was injected with 1.0% DMSO (vehicle) or 6OTD (10 or 100 pmol) intracranially on the same day as the GSC transplantation. n = 5 (vehicle) or 6 (6OTD-treated). Immunohistochemistry of mouse brains stained with human-specific anti-vimentin (top) or anti-nestin (bottom) antibodies. Original magnification of the images was 4×. Graphs indicate the relative tumor size as determined by immunohistochemistry with the glioma markers described above. Error bars indicate the SD values (**P < 0.01). (**B**) Body weight change of vehicle and 6OTD- (10 or 100 pmol) treated groups.

Finally, we examined the antitumor activity of 6OTD against GSCs *in vivo*. Mice were injected intracranially with GBM146 neurospheres to establish GSC-orthotopic transplanted mice at day 0. On the same day, we then administered 10 or 100 pmol of 6OTD into the same space as the GSCs. Mice were sacrificed at 3 months after transplantation and their brains removed for further evaluation. Immunohistochemistry was performed on brain sections using human-specific antibodies to vimentin and nestin. Vimentin staining revealed 70% and 79% reductions in overall tumor volumes were observed following treatment with 10 and 100 pmol 6OTD, respectively (Fig. 6A, top). Similarly, nestin staining revealed that 6OTD treatment led to 60% (10 pmol) and 81% (100 pmol) reductions in tumor size (Fig. 6A, bottom). We also measured the body weight of mice from day 0 to when mice were sacrificed. 6OTD did not have any significant effects on mouse body weight (Fig. 6B). Additionally, no behavioral differences were observed in drug-treated mice as compared with vehicle-treated mice. These observations indicate that 6OTD suppressed the intracranial growth of GSCs in a dose-dependent manner without any significant side effect.

## Discussion

We have evaluated the growth inhibitory activity of 6OTD, a TMS derivative with gram-scale availability, against glioblastoma and GSCs both *in vitro* and *in vivo*. 6OTD preferentially inhibited the growth of GSCs at lower concentrations than did TMS. This synthetic G4 ligand induced G1-arrest of the cell cycle, apoptosis, and DNA damage responses presumably on G4-forming sequences in GSCs. In intraperitoneally and intracranially xenografted mouse therapeutic models, no obvious side effects, such as behavioral abnormality or weight loss, were observed irrespective of the route of 6OTD administration (Figs 2 and 6).

FRET melting assay analysis revealed that 6OTD has lower *ΔT*_m_ values for telomeric and *bcl*-*2* GFOs than does TMS. We initially assumed that the G4-stabilizing activities of G4 ligands would correlate with their inhibitory effects on cell growth. Unexpectedly, evaluation of the JFCR39 human cancer cell line panel revealed that 6OTD has a greater anti-proliferative activity than TMS: the GI_50_ values of 6OTD were lower than those of TMS in 35 of 39 cell lines. Similarly, 6OTD more efficiently suppressed the growth of GSCs than did TMS. We cannot rule out the possibility that cell membrane permeability between these two G4 ligands is different; that is, 6OTD may penetrate the cell membrane more effectively than TMS. We have not investigated this issue because of the limited availability of TMS. Another possible explanation is that 6OTD-preferred G4s may not be included in the GFOs tested in the FRET melting assay. A recent study suggests that humans have more than 500,000 G4-forming motifs in their genome^46^. Therefore, we suspect that other G4 sequences, whose stabilization effectively suppresses the growth of GSCs, are targeted by 6OTD but not TMS. We speculate that the target diversity of G4 ligands could be linked to the different sensitivity patterns of 6OTD and TMS.

G4 targeting as a potential cause of the biological effect on GSCs is supported by several observations: (i) 6OTD induced telomeric DNA damage more frequently than the alkylating agent TMZ in GSCs; (ii) other G4-stabilizing agents, TMS and Phen-DC3, also inhibited the growth of GSCs more potently than NSGCs^12, 21^ (S.O., unpublished observation); (iii) BIBR1532, a telomerase inhibitor without G4-stabilizing activity, does not cause a preferential blockade of GSC growth^21^. Meanwhile, molecular profiling analyses indicate that 6OTD does not inhibit EGFR, MET, ABL, FLT-1, ALK, IGF-1R, RET, TrkA, AKT, S6, ERK, PLCγ1, or PKD kinases, HDAC1, HDAC6, or SIRT1 histone deacetylases, indicating that these oncogenic factors, at least, would not be the targets of 6OTD (K.N. and H.S., unpublished observations). These observations suggest G4 stabilization, rather than inhibition of telomerase or other typical oncogenic factors, as the primary cause of the biological effect on GSCs.

What then is the molecular mechanism underlying GSC-selective growth inhibition by 6OTD? Given that G4s may modulate various biological events, including replication, transcription, and translation, it is possible that G4 ligand action could also be influenced by these events. Consistently, we and other groups have reported that the DNA damage response induced by G4 ligands depends on replication and transcription^21, 47^. Accordingly, the higher abundance of EdU-positive (i.e., DNA synthesizing) cell fractions in GSCs compared with NSGCs may partly explain the higher sensitivity of GSCs to TMS and 6OTD. Another possible explanation is that GSCs express higher levels of replication stress proteins. Therefore, G4 ligands may exert a preferential antitumor effect on GSCs dependent on potent activation of the ATR–Chk1 replication stress and DNA damage response pathways^21^. Meanwhile, GSCs express higher levels of c-*myb*, downregulation of which contributes to the GSC-selective growth inhibition by TMS^12^. Similarly, 6OTD downregulates c-*myb* expression only in GSCs (S.O., unpublished observation). This phenomenon could also be involved in GSC-selective cell growth inhibition. Simply put, the molecular targets of 6OTD would ordinarily be responsible for GSC survival.

We have not obtained pharmacokinetic data for 6OTD, and whether 6OTD can penetrate the blood–brain barrier remains unknown. Therefore, further development of this G4 ligand or its advanced compound as an effective anticancer agent for GBM will require that the dosage form and drug delivery system be optimized to enable access to brain tissue. Another issue concerning the efficacy of 6OTD against GSCs is that this compound, at least in the present treatment protocol, cannot completely eliminate all GSCs *in vivo*. We presume that a single intracranially-administered dose of 6OTD is insufficient to eradicate GSCs. Additionally, 6OTD-resistant cells in the heterogeneous population of GSCs may also be responsible for disease relapse. Further pharmacokinetic data, such as blood and tumor tissue concentrations of the compound, and identification of pharmacodynamic markers of 6OTD, will allow higher-resolution monitoring of xenograft tumors and facilitate the development of G4 ligands as an innovative anti-cancer medicine.

In summary, exposure of GSCs to 6OTD, which stabilizes G4s in telomeres and several oncogene promoter sequences *in vitro*, induces a DNA damage response and ultimately apoptosis. These observations suggest that G4s may represent a promising therapeutic target for GSCs. Further exploration of crucial target G4s and a detailed understanding of the growth inhibitory mechanisms by which 6OTD acts against GSCs may reveal new means to treat glioblastoma. Identifying potential biomarkers of sensitivity or resistance to TMS derivatives will also be useful for selecting patients likely to respond to these compounds.

## Methods

### Chemicals

6OTD was synthesized and its chemical identity was established as previously reported^31^. TMS was prepared as previously reported^11^ and dissolved at 5 mM in a 1:1 solution of DMSO/methanol (both from Nacalai Tesque, Kyoto, Japan). TMZ was purchased from Sigma-Aldrich (St. Louis, MO, USA) and dissolved in DMSO.

### FRET melting assay

The dual fluorescence-labeled oligonucleotides telo21 [5′-FAM-d(GGGTTAGGGTTAGGGTTAGGG)-TAMRA-3′], *bcl*-*2* [5′-FAM-d(GGGCGCGGGAGGAAGGGGGCGGG)-TAMRA-3′], *c*-*kit* [5′-FAM-d(GGGAGGGCGCTGGGAGGAGGG)-TAMRA-3′], *c*-*myc* [5′-FAM-d(GAGGGTGGGGAGGGTGGGGAAG)-TAMRA-3′], *k*-*ras* [5′-FAM-d(AGGGCGGTGTGGGAAGAGGGAAGAGGGGGAGG)-TAMRA-3′], and dsDNA [5′-FAM-d(TATAGCTATATTTTTTTATAGCTATA)-TAMRA-3′] were purchased from Sigma Genosys (Spring, TX, USA)^32^. All purified nucleotides were dissolved as a 10 μM stock solution in MilliQ water. Further dilutions of the oligonucleotides were performed with FRET buffer [60 mM potassium cacodylate buffer (pH 7.4), Wako, Osaka, Japan], and dual-labeled DNAs at 400 nM were annealed by heating at 96 °C for 2 min followed by cooling to room temperature. 6OTD (10 mM) and TMS (5.0 mM) were diluted to 200 μM using DMSO. These ligands were further diluted to 2.0 μM with FRET buffer. The annealed DNA and the compound solutions (20 μL of each) were distributed across 96-well plates (Roche Life Science, Penzberg, Germany) for a total reaction volume of 40 μL, with 200 nM of labeled oligonucleotide and 1.0 μM of the compounds. These mixtures were incubated at 4 °C overnight. Measurements were carried out in triplicate with an excitation wavelength of 470 nm and a detection wavelength of 514 nm using a LightCycler® 96 Real-Time PCR System (Roche Life Science). The change in melting temperature at a 1.0 μM ligand concentration [*ΔT*_m_ (1.0 μM)] was calculated from three experiments by subtraction of the blank from the averaged 1.0 μM ligand melting temperature.

### CD spectroscopy and melting assay

A solution of the oligonucleotides was prepared in 50 mM Tris-HCl with 100 mM KCl at concentration of 10 μM and annealed at 99 °C for 5 min, then cooled down to room temperature for overnight. CD spectra were recorded on a J-720 spectropolarimeter (JASCO, Tokyo, JAPAN) using a quartz cell of 1 mm optical path length and an instrument scanning speed of 500 nm/min with a response time of 1 s, and over a wavelength ranger of 220–320 nm. Finally CD spectra are representative of five averaged scans taken at 25 °C, then a stepwise increase of 10 °C from 25 °C to 95 °C. Melting curve was obtained by plotting the CD intensity at 295 nm, and *T*_*m*_ values were determined by fitting the melting curve using ImageJ software.

### Cell lines and cell culture

Human glioblastoma U251 cells and GSC lines GBM146 and GBM157^48^ were maintained as described in Supplementary Information. GSCs were differentiated into NSGCs by seeding into the adherent culture medium (DMEM/F-12 supplemented with 10% FBS, 1% penicillin-streptomycin, and 0.1 mg/mL kanamycin sulfate). NSGCs were used for subsequent experiments 7 days after cultivation in the adherent culture medium.

### *In vivo* efficacy in U251 mouse xenografts

All animal procedures were performed in the animal experiment room of the Japanese Foundation for Cancer Research (JFCR) according to protocols approved by the JFCR Animal Care and Use Committee. Approximately 1.0 × 10^7^ U251 cells were resuspended in 100 μL of Hanks’ balanced salt solution (Thermo Fisher Scientific) and then subcutaneously implanted into the right flanks of 6-week-old CAnN. Cg-Foxn1nu/CrlCrlj nude mice (Charles River Laboratories Japan, Kanagawa, Japan). The length (*L*) and width (*W*) of the tumor mass were measured by caliper to determine the tumor volumes (*TV*) calculated as

$$TV=(L\times {W}^{2})/2.$$

Eighteen days after implantation (=day 0), xenotransplant mice were randomized into control and treatment groups (n = 6 per group). Mice in the treatment group were administered 240 m/kg of 6OTD in 10% DMSO/saline five times a week (day 0–4, 7–11, 14–18, 21–25, 28–32, 35–39) and the control mice were administered 10% DMSO/saline on the same days as the treated mice after measurement of their tumor volumes and body weights. On day 39, all mice were sacrificed and tumors were excised and weighed.

### Cell proliferation assay

Approximately 1.0 × 10^4^ cells in 800-μL relevant culture medium were seeded into each well of a 24-well microplate for floating culture (GSCs) or adherent culture (NSGCs) and incubated for 16 h. These cells were treated with 200 μL of TMS or 6OTD at increasing concentrations and further incubated for 6 days. Subsequently, these cells were detached with 200 μL of TrypLE™ Select containing 10 mM EDTA (Nacalai Tesque), incubated for 10 min at 37 °C, and then resuspended in 9.8 mL of Cell pack (Sysmex, Hyogo, Japan). The number of cells in each well was counted with a CDA-500 (Sysmex).

### Cell cycle analysis

Cells were treated with DMSO or 6OTD (100 nM) 1 day after seeding. These cells were incubated for 5 days and washed with phosphate-buffered saline (PBS) followed by a centrifugation at 4 °C for 5 min at 210 × *g*. Cell pellets were resuspended in 900 μL of PBS, and cells were subsequently fixed in 2.1 mL of ice-cold ethanol. After incubation for 30 min at 4 °C followed by a centrifugation at 4 °C for 5 min at 1,920 × *g*, cell pellets were washed with PBS. Next, cell pellets were resuspended in 400 μL of 2.0 mg/mL RNase A (Sigma-Aldrich), followed by 30 min incubation at 37 °C and washing with PBS. The cell pellets were resuspended in 1.0 mL of 50 μg/mL PI (Sigma-Aldrich) and incubated for 15 min in the dark at room temperature. After filtration through 35-μm nylon mesh (BD), these cells were analyzed using a FACS Calibur (BD).

### Western blot analysis

Cells were resuspended in whole-cell-extract buffer consisting of 150 mM NaCl, 1.0% NP-40, and 50 mM Tris-HCl (pH 8.0) along with protease inhibitor cocktail (1:50, Nacalai Tesque) and 0.125 mM dithiothreitol (Sigma-Aldrich) and left on ice for 30 min with vortexing every 10 min. After centrifugation at 12,000 rpm for 10 min at 4 °C, the supernatants were collected as whole cell extracts and subjected to SDS-PAGE. Separated proteins were then transferred to polyvinylidene difluoride membranes (Merck Millipore). These membranes were blocked with 5.0% w/v nonfat dry milk in Tris-buffered saline (TBS: 10 mM Tris base, 40 mM Tris-HCl, and 150 mM NaCl) containing 0.1% Tween-20 (TBST) for 1 h at room temperature, and incubated with the required primary antibody in TBST containing 5.0% w/v nonfat dry milk. After washing in TBST, the membranes were incubated with horseradish peroxidase-conjugated anti-mouse or anti-rabbit IgG in TBST containing 5.0% w/v nonfat dry milk for 1 h at room temperature. After extensive washing with TBST, signals were visualized using an ECL Western Blotting Detection System (RPN2106, GE Healthcare) or Pierce ECL Plus Western Blotting Substrate (NCI32132, Thermo Fisher Scientific).

### Immunofluorescence staining and iFISH

Cells were fixed with 2% (w/v) paraformaldehyde/PBS and subjected to immunofluorescence staining as described previously^21^. iFISH was performed essentially as described^49^. Detailed methods for both experiments are described in Supplementary Information.

### Intracranial mouse xenografts

Approximately 1.0 × 10^5^ GSCs were resuspended in 10 μL of Hanks’ balanced salt solution and transplanted into the brains of CAnN. Cg-Foxn1nu/CrlCrlj nude mice (Charles River Laboratories Japan, Kanagawa, Japan). Solutions (1 μL) containing DMSO or 6OTD (10 and 100 pmol) diluted in Hanks’ balanced salt solution were then injected over 1 min into the same location as the GSCs. Three months after injection, all mice were sacrificed and their brains excised.

### Immunohistochemistry

Paraffin-embedded sections were deparaffinized by dipping into xylene. To substitute ethanol for xylene, sections were dipped into 70% ethanol and distilled water. Antigen activation was conducted by incubating samples for 15 min at 100 °C, then for 30 min at room temperature using DAKO REAL Target Retrieval Solution (DAKO, Glostrup, Denmark). After washing with TBST and distilled water, all sections were dipped in 0.3% hydrogen peroxide in methanol for 10 min at room temperature. Samples were then washed with running water and TBST, followed by blocking with TBST containing 10% goat serum for 30 min at room temperature. Subsequently, all sections were incubated with the required primary antibodies in TBST containing 10% goat serum for 14 h at 4 °C. After washing with TBST, samples were stained with DAKO REAL EnVision Detection System (DAKO) according to manufacturer’s instructions. After washing with running water and TBST, all slides were stained with hematoxylin for 5 sec, followed by washing with running water and distilled water. These sections were dehydrated with 70% and then 100% ethanol, subsequently, and substituted with xylene. Finally, samples were sealed with Entellan New (Merck Millipore) and images were acquired using an IX83 instrument (Olympus). These images were imported into ImageJ software and the color channels were deconvoluted into DAB and hematoxylin. The intensities of DAB immunostaining were measured automatically with the same software.

### Antibodies

Antibodies used in this study are described in Supplementary Information.

## Electronic supplementary material

Supplementary Information
