# Supplementary material for: Targeting glioma stem cells in vivo by a G-quadruplex-stabilizing synthetic macrocyclic hexaoxazole
Source: Sci Rep. 2017 Jun 15;7:3605. doi: 10.1038/s41598-017-03785-8 (PMC5472576; doi:10.1038/s41598-017-03785-8)
Supplement: Supplementary file 1 — Supplementary Information [file 41598_2017_3785_MOESM1_ESM.pdf]

**Supplementary Information**

# Targeting glioma stem cells *in vivo* by a G-quadruplex-stabilizing synthetic macrocyclic hexaoxazole

Takahiro Nakamura, Sachiko Okabe, Haruka Yoshida, Keisuke Iida, Yue Ma, Shogo Sasaki, Takao Yamori, Kazuo Shin-ya, Ichiro Nakano, Kazuo Nagasawa and Hiroyuki Seimiya


- **Supplementary Methods**
- **Supplementary References**
- **Supplementary Table S1**
- **Supplementary Figure S1**
- **Supplementary Figure S2**
- **Supplementary Figure S3**

## Supplementary Methods

### Surface plasmon resonance (SPR) assay.

Surface plasmon resonance (SPR) measurements were performed with a four-channel BIAcore T200 instrument (GE Healthcare) and streptavidin-coated sensor chip (Biacore SA-chip). All measurements were performed at 25°C using a running buffer (HEPES buffer), which was prepared using 10 mM HEPES (pH 7.4), 150 mM NaCl, 3 mM EDTA and 0.005% surfactant P20. We have used two biotinylated DNA; telo24 (5'-biotin-d[TTAGGG]$_4$-3') and dsDNA (5'-biotin-d[TATAGCTATATTTTTTTATAGCTATA]-3'), and all DNAs were denatured in HEPES buffer at 99°C for 5 min, then allowed to cool down to room temperature for overnight. The flow cells 2 and 4 were used to immobilize the DNA, while the 1 and 3 flow cells were left blank as a control. After conditioning the surface with three 1 min injected of 1 M NaCl/50 mM NaOH, a solution of DNA samples (100 nM in running buffer) was injected at a rate of 10 µL/min. For binding experiments, a stock solution of Y2H2-6M(4)OTD (6OTD) was prepared at a 10 µM concentration in running buffer, and experimental concentration was prepared in the desired by serial dilutions from stock solution. The experimental solutions at concentrations from 2 to 1000 nM were injected through the DNA and blank flow cells at a rete of 30 µL/min. To remove any remaining bound compound after the dissociation phase of the sensorgram, a low pH glycine regeneration buffer was used (10 mM glycine at pH 2.0). The baseline was then reestablished, and the next compound concentration sample was injected. For 6OTD dissociation constants was determined by fitting the binding curve using BIAevaluation software (GE Healthcare).

### Cell lines and cell culture.

Human glioblastoma U251 cells were cultured in RPMI medium 1640 (Thermo Fisher Scientific) supplemented with 10% (v/v) heat-inactivated fetal bovine serum (FBS; Cellgro, Tewksbury, MA, USA), 1% (v/v) penicillin-streptomycin (Thermo Fisher Scientific), and 0.1 mg/mL kanamycin sulfate (Meiji Seika Pharma, Tokyo, Japan). GSC lines derived from human glioblastoma GBM146 and GBM157 [1] were maintained in the sphere medium [Dulbecco's modified Eagle Medium (DMEM)/F-12, HEPES (Thermo Fisher Scientific), 1%

penicillin-streptomycin, 0.1 mg/mL kanamycin sulfate], supplemented with B-27, Glutamax (Thermo Fisher Scientific), 5.0 μg/mL heparin (Sigma-Aldrich), 20 ng/mL basic fibroblast growth factor (bFGF; PeproTech, Rocky Hill, NJ, USA), and 50 ng/mL epidermal growth factor (EGF; PeproTech) using flasks for floating culture (Asahi glass, Tokyo, Japan). Glutamax, 20 ng/mL bFGF, and 50 ng/mL EGF were added to cultures twice a week. These neurospheres were transferred into fresh medium after dissociation by TrypLE™ Express (Thermo Fisher Scientific) once a week.

**Antibodies.**

Rabbit anti-cleaved PARP (Asp214, #9541) and rabbit anti-53BP1 (#4935) were obtained from Cell Signaling Technology (Danvers, MA, USA). Mouse anti-γH2AX (monoclonal, 2506483) and mouse anti-nestin (monoclonal, #MAB5326) were from Merck Millipore (Darmstadt, Germany). Rabbit anti-vimentin (monoclonal, ab92547) was from Abcam (Cambridge, UK). Mouse anti-PARP (556362) was from Becton, Dickinson and Company (Franklin Lakes, NJ, USA). Mouse anti-β-actin (A5441) was from Sigma-Aldrich. As secondary antibodies, Alexa Fluor® 594-conjugated anti-mouse IgG (H+L) (A-11032) and Alexa Fluor® 488-conjugated anti-rabbit IgG (H+L) (A-11034) were obtained from Thermo Fisher Scientific (Waltham, MA, USA). Peroxidase-linked anti-mouse IgG (NA931V) and anti-rabbit IgG (NA934V) were obtained from GE Healthcare (Milwaukee, WI, USA).

**Immunofluorescence staining.**

GSCs and NSGCs in the relevant culture media (400 μL/well) were seeded into 12-well microplates on top of poly-L-lysine-coated coverslips (Matsunami Glass, Osaka, Japan) in 400 μL/well of the adherent culture medium. After incubation for 3 h (GSCs) or 24 h (NSGCs), each well was washed with sphere culture medium and 6OTD or TMZ in the sphere culture medium containing all supplements were added into each well. After incubation for 3 days, cells were fixed with 2% (w/v) paraformaldehyde/PBS, permeabilized with Nonidet P-40/PBS, and blocked with 1% (w/v) bovine serum albumin (BSA)/PBS as described previously [2]. Each coverslip was incubated with anti-53BP1 (1:100)

and anti-γH2AX (1:4,000) antibodies for 1 h at room temperature, and subsequently washed with 1% (v/v) BSA/PBS. After incubation with Alexa Fluor® 488-conjugated anti-rabbit IgG (1:500) and Alexa Fluor® 594-conjugated anti-mouse IgG (1:500) in 1% BSA/PBS, coverslips were washed with 1% BSA/PBS and placed onto Vectashield medium containing 4',6-diamidino-2-phenylindole (DAPI; Vector laboratories, Burlingame, CA, US) on slide glasses. Images were captured with a fluorescence microscope IX71 and Dual-CCD DP80 digital camera (Olympus, Tokyo, Japan).

**Immunofluorescence *in situ* hybridization (iFISH).**

Cells on coverslips were fixed and permeabilized as described above. Then the cells were dehydrated by 5-min incubations with 100 μL each of 70%, 95%, and 100% EtOH. After 10 min of air-drying, coverslips were transferred onto 10 μL of hybridization mix added dropwise on glass slides and incubated for 5 min at 80°C and then 1 h at room temperature. Coverslips were then washed with washing buffer and with 1% BSA/PBS. Subsequently, non-specific sites were blocked with 1% BSA/PBS and incubated with rabbit anti-53BP1 (Cell Signaling Technology, 1:100) for 1 h at room temperature. Washed coverslips were then incubated with Alexa Fluor 488-conjugated anti-rabbit IgG (1:500) in 1% BSA/PBS for 30 min at room temperature and extensively washed with 1% BSA/PBS. Coverslips were placed onto 10 μL of Vectashield medium containing DAPI added dropwise on glass slides. Images were captured as described above or with a confocal microscope FV1000 (Olympus).

**Supplementary References**

1 Visnyei, K. *et al.* A molecular screening approach to identify and characterize inhibitors of glioblastoma stem cells. *Mol Cancer Ther* **10**, 1818-1828 (2011).

2 Hasegawa, D. *et al.* G-quadruplex ligand-induced DNA damage response coupled with telomere dysfunction and replication stress in glioma stem cells. *Biochem Biophys Res Commun* **471**, 75-81 (2016).

**Supplementary Table S1 | $IC_{50}$ values of 6OTD against JFCR39 human cancer cell line panel**

| | Cell line | $IC_{50}$ value (mol/L) |
|---|---|---|
| Breast | HBC-4 | 5.70E-08 |
| | BSY-1 | 6.20E-08 |
| | HBC-5 | 3.80E-07 |
| | MCF-7 | 5.50E-05 |
| | MDA-MB-231 | >1.0E-04 |
| CNS | U251 | 2.10E-08 |
| | SF-268 | 3.70E-08 |
| | SF-295 | 2.90E-07 |
| | SF-539 | 8.70E-08 |
| | SNB-75 | 1.60E-07 |
| | SNB-78 | 1.80E-07 |
| Colon | HCC2998 | 9.90E-08 |
| | KM-12 | 5.20E-08 |
| | HT-29 | 1.30E-07 |
| | HCT-15 | >1.0E-04 |
| | HCT-116 | 9.20E-08 |
| Lung | NCI-H23 | 1.30E-07 |
| | NCI-H226 | 6.90E-08 |
| | NCI-H522 | 6.10E-08 |
| | NCI-H460 | 2.60E-07 |
| | A549 | 5.70E-07 |
| | DMS273 | 1.10E-07 |
| | DMS114 | 1.10E-07 |
| Melanoma | LOX-IMVI | >1.0E-04 |
| Ovarian | OVCAR-3 | 1.20E-07 |
| | OVCAR-4 | 2.40E-07 |
| | OVCAR-5 | 7.90E-07 |
| | OVCAR-8 | 3.50E-07 |
| | SK-OV-3 | 3.00E-07 |
| Renal | RXF-631L | 1.90E-07 |
| | ACHN | 1.40E-07 |
| Stomach | St-4 | 2.10E-07 |
| | MKN1 | 1.40E-07 |
| | MKN7 | 4.50E-08 |
| | MKN28 | 6.70E-08 |
| | MKN45 | 1.10E-07 |
| | MKN74 | 1.20E-07 |
| Prostate | DU145 | 2.20E-07 |
| | PC-3 | 1.30E-07 |

Cells were treated with various concentrations of 6OTD for 2 days and $IC_{50}$ values were calculated.

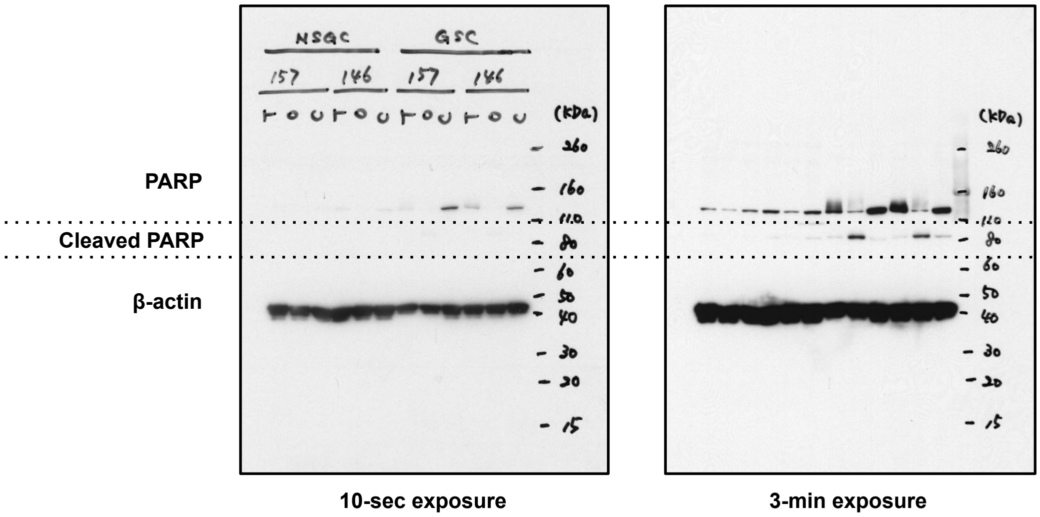


**Supplementary Figure S1 | Full-length western blots for Figure 3(E)**

Glioma stem cells (GSCs) and non-stem glioma cells (NSGCs) were lysed 5 days after DMSO, 6OTD (100 nM), or TMZ (10 μM) treatment, and the resulting lysates were subjected to SDS-PAGE. Separated proteins in the gel were transferred to a polyvinylidene membrane, which was cut into three pieces at the dotted lines. The *upper*, *middle*, and *bottom* membranes shown in the figure were incubated with anti-PARP, cleaved-PARP, and β-actin antibodies, respectively. Blots with short (10 sec, *left*) and long (3 min, *right*) exposure are shown. PARP/cleaved PARP and β-actin blots were cropped with Adobe Photoshop CS5 from the long and short exposure images, respectively, and their mirrored images were demonstrated in Figure 3(E). C: control, O: 6OTD, T: TMZ, 146: GBM146, 157: GBM157.

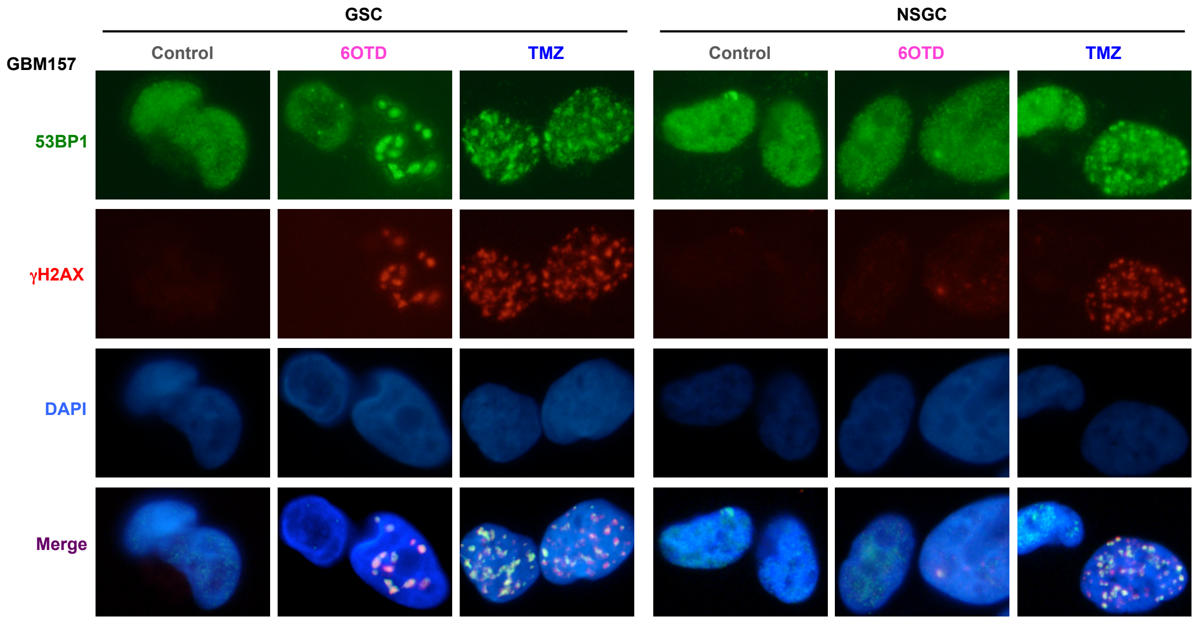


**Supplementary Figure S2 | DNA damage responses in GBM157 GSCs and NSGCs exposed to 6OTD and TMZ**

GBM157 GSCs and NSGCs were treated with DMSO, 6OTD (100 nM), or TMZ (10 μM) for 3 days and subjected to indirect immunofluorescence staining with anti-53BP1 (green) and anti-γH2AX (red) antibodies as described in *Methods*. Nuclear DNA was counterstained with DAPI (blue).

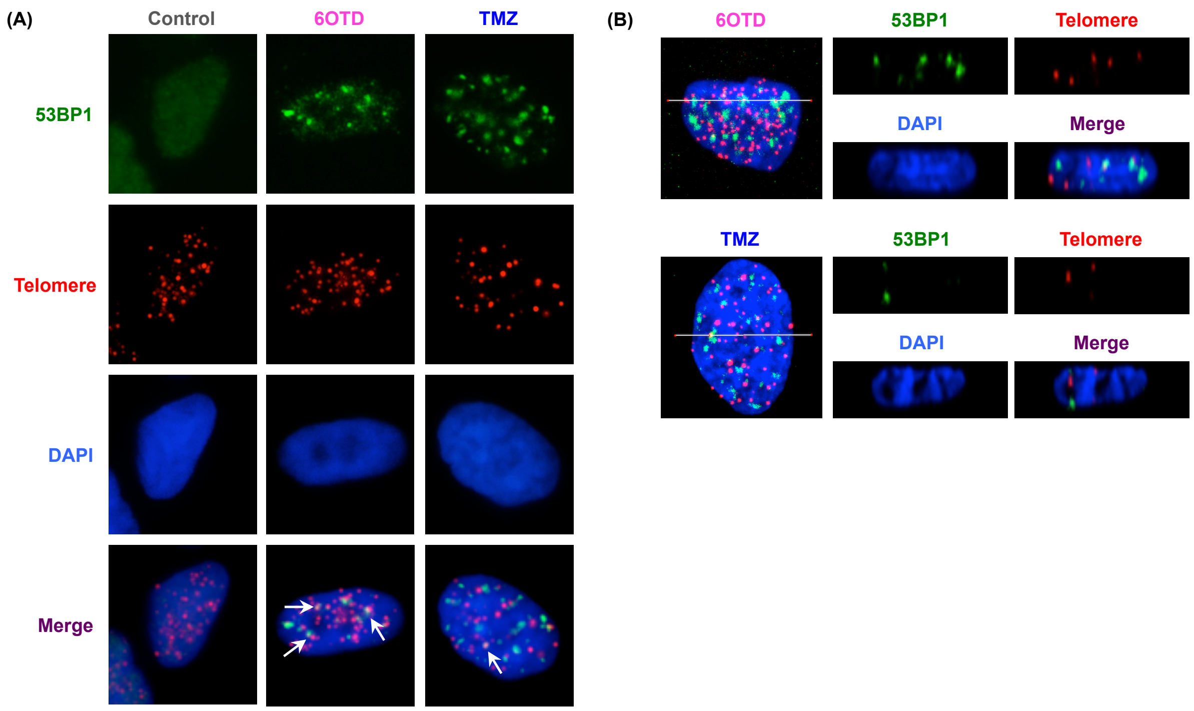


**Supplementary Figure S3 | iFISH analysis for evaluating telomere dysfunction-induced foci (TIF) in GBM157 GSCs treated with 6OTD and TMZ**

(A) GBM157 GSCs were treated with DMSO, 6OTD (100 nM), or TMZ (10 μM) for 3 days and subjected to iFISH analysis with anti-53BP1 antibody (green) and PNA probe for G-rich telomeric DNA (red) as described in *Methods*. *White arrows* indicate TIFs (*bottom panels*).

(B) *Z*-stack image of 6OTD (*upper*) or TMZ (*lower*)-treated GSCs and consecutive *xz* images on white lines of those cells labeled.
